# Autophagy is a new protective mechanism against the cytotoxicity of platinum nanoparticles in human trophoblasts

**DOI:** 10.1038/s41598-019-41927-2

**Published:** 2019-04-02

**Authors:** Akitoshi Nakashima, Kazuma Higashisaka, Tae Kusabiraki, Aiko Aoki, Akemi Ushijima, Yosuke Ono, Sayaka Tsuda, Tomoko Shima, Osamu Yoshino, Kazuya Nagano, Yasuo Yoshioka, Yasuo Tsutsumi, Shigeru Saito

**Affiliations:** 1 Department of Obstetrics and Gynecology, University of Toyama, 2630, Sugitani, Toyama, 930-0194 Japan; 2 Laboratory of Toxicology and Safety Science, Graduate School of Pharmaceutical Sciences, Osaka University, 1-6 Yamadaoka, Suita, Osaka 565-0871 Japan; 3 Department of Legal Medicine, Graduate School of Medicine, Osaka University, 2-2 Yamadaoka, Suita, Osaka 565-0871 Japan; 4 Vaccine Creation Project, BIKEN Innovative Vaccine Research Alliance Laboratories, Research Institute for Microbial Diseases, Osaka University, 3-1 Yamadaoka, Suita, Osaka 565-0871 Japan; 5 BIKEN Center for Innovative Vaccine Research and Development, The Research Foundation for Microbial Diseases of Osaka University, 3-1 Yamadaoka, Suita, Osaka 565-0871 Japan; 6 The Center for Advanced Medical Engineering and Informatics, Osaka University, 1-6, Yamadaoka, Suita, Osaka 565-0871 Japan; 7 Department of Obstetrics and Gynecology, Kitasato University School of Medicine, 1-15-1 Kitazato, Minami, Sagamihara, Kanagawa 252-0374 Japan

## Abstract

Nanoparticles are widely used in commodities, and pregnant women are inevitably exposed to these particles. The placenta protects the growing fetus from foreign or toxic materials, and provides energy and oxygen. Here we report that autophagy, a cellular mechanism to maintain homeostasis, engulfs platinum nanoparticles (nPt) to reduce their cytotoxicity in trophoblasts. Autophagy was activated by nPt in extravillous trophoblast (EVT) cell lines, and EVT functions, such as invasion and vascular remodeling, and proliferation were inhibited by nPt. These inhibitory effects by nPt were augmented in autophagy-deficient cells. Regarding the dynamic state of nPt, analysis using ICP-MS demonstrated a higher accumulation of nPt in the autophagosome-rich than the cytoplasmic fraction in autophagy-normal cells. Meanwhile, there were more nPt in the nuclei of autophagy-deficient cells, resulting in greater DNA damage at a lower concentration of nPt. Thus, we found a new protective mechanism against the cytotoxicity of nPt in human trophoblasts.

## Introduction

Pregnant women and developing fetuses are very susceptible to foreign toxins, including air pollutants, microbes, and nanoparticles^1–3^. Smaller nanoparticles made of silica, titanium dioxide, cobalt and chromium, gold, or silver cross the fetal-maternal barrier more readily than larger particles^4–7^. Recently, exposure to nanoparticles in the gestational period is becoming a public concern because it may cause developmental disorders in the offspring. However, nanoparticles are currently used in a variety of consumer products such as foods, cosmetics, electronics, and drug delivery systems^8–12^. Among the metallic nanoparticles, platinum nanoparticles as well as silver nanoparticles have potentially detrimental effects on cells, organs, and bacteria^13–17^. However, cellular responses evoked by nanoparticles differ according to the properties or modifications of each nanoparticle.

The placenta functions in nutrient and oxygen exchange between the mother and fetus, as well as in protection of the fetus from harmful materials^18^. Extravillous trophoblast (EVT) cells invade the myometrium or maternal spiral arteries under low oxygen conditions, and replace the endothelial cells to supply oxygen and nutrition to the intervillous space^19^. Placental insufficiency or poor placentation, which is related to insufficient invasion of EVT cells into the maternal side^19^, causes severe pregnancy complications such as preeclampsia, fetal growth restriction, or placental abruption^20,21^. Among these small potentially hazardous particles, differently sized and typed nanoparticles that cross the placenta can reach the fetal brain, resulting in neurodevelopmental abnormalities^5,7,22^. In particular, silica nanoparticles (nSP) accumulate in the liver and placenta in pregnant mice, and nSP with a diameter of 70 nm are specifically trapped in the placenta, but not the liver^7^. Administration of 70-nm nSP also induced the inflammasome components, resulting in placental inflammation, which is known to cause pregnancy complications such as preeclampsia and preterm labor^19,23^. In addition, not only intravenously, but also orally administered silver nanoparticles, which are eluted in breast milk during lactation, were distributed in the brain, liver, and lungs in the fetus^24^. To reduce the risks of nanoparticles for mothers and fetuses, it is important to evaluate the mechanism by which these nanoparticles confer cytotoxicity to the placenta.

Autophagy is a cellular mechanism for maintaining homeostasis by degrading damaged organelles or countervailing a variety of detrimental agents, including intrusion of foreign micro-organisms, i.e., xenophagy^25^. There is increasing evidence regarding the correlation between autophagy and nanoparticles; there are some reports of nanoparticle-activated autophagy^26–28^, whereas others reported inhibition of autophagy^4,29,30^. It is unknown, however, how designed nanoparticles interact with the autophagy pathway in detail^31^. From the viewpoint of autophagic functions for nanoparticles, autophagy protects cells from internalized nanoparticles, which exert toxicity through oxidative stress^32^, mitochondrial damage^33^, lysosomal dysfunction^34^, or direct inhibition of the AKT-TSC-mTOR pathway^35^. In particular, the biodegradability and surface modification of nanoparticles affected the lysosomal stability in a hepatocellular cell line, resulting in several cellular process being altered via mTOR regulation^36^. On the other hand, silver nanoparticles have negative effects on autophagy by inhibiting autophagosome-lysosome fusion^29^.

We report that platinum nanoparticles (nPt), which are one nanometer in size, activated autophagy in two extravillous trophoblast (EVT) cell lines. nPt also impaired the functions, such as invasion and vascular remodeling, and proliferation of EVT cell lines, and this impairment was reduced in autophagy-deficient cells. After separating autophagosome-rich and cytoplasmic fractions, nPt were accumulated in the autophagosome-rich fraction, resulting in the reduction of cytotoxicity by nPt. Meanwhile, nPt, which were not trapped by autophagosomes, was highly accumulated in nuclei of autophagy-deficient cells, showing more susceptible to DNA damage by nPt. Thus, autophagy protected against the cytotoxicity of nPt in the EVT cell lines.

## Results

### Autophagy activation by nPt in two EVT cell lines

**Figure 1:**
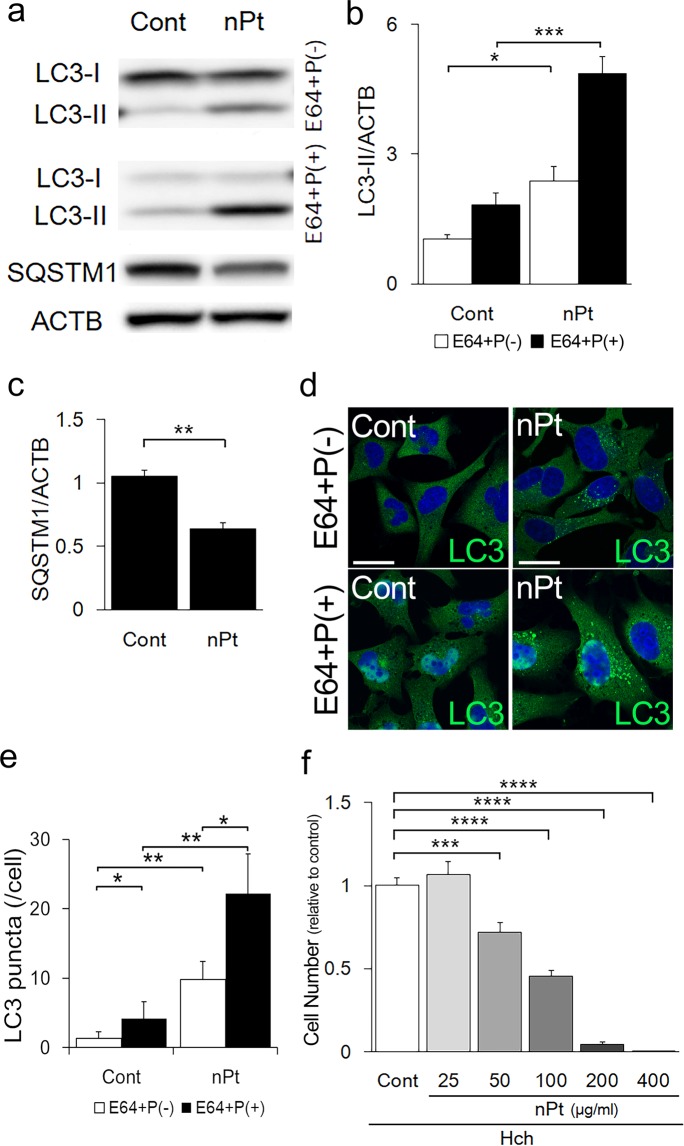
Autophagy activation by nPt in HchEpC1b cells, an EVT cell line. (**a**) Western blots of HchEpC1b cells, which were cultured with 25 μg/ml of nPt for 24 h with or without E64d (E64) and pepstatin A (P) for 2 h at the end of culture, were as follows: MAP1LC3B (LC3), SQSTM1, and ACTB. The expression levels of MAP1LC3B-II (**b**) or SQSTM1 (**c**) in the HchEpC1b cells cultured with nPt were shown in the graphs. Expression was normalized with ACTB levels. (**d**) Representative panels showed the merged images of anti-MAP1LC3B staining (LC3, green) and nuclei staining (DAPI, blue) of HchEpC1b cells cultured with 25 μg/ml of nPt for 24 h in the presence or absence of E64d and pepstatin for 2 h. (**e**) The graph showed the average number of LC3 puncta in HchEpC1b cells treated with the presence (black bars) or absence (white bars) of E64 and P, as shown in (**d**). (**f**) The cell number of HchEpC1b cells with nPt at the indicated concentrations (μg/ml) for 24 h were shown. The numbers of cells in the treatment groups were normalized to that with control treatment, PBS, as one. Data were expressed as the mean ± S.D. *p < 0.05, **p < 0.01, ***p < 0.001. Scale bars: 20 μm.

We first evaluated the effects of nPt on autophagy in EVT cells. nPt promoted the conversion of MAP1LC3B-I to MAP1LC3B-II, and decreased SQSTM1/p62 levels, a substrate of the autophagosome, in HchEpC1b cells, an EVT cell line (Fig. 1a). The MAP1LC3B-II/ACTB levels were significantly higher in the cells cultured with nPt than in the control in the presence or absence of E64d and pepstatin A (E64 + P), which block autophagy flux by inhibiting lysosomal proteases^37^ (Fig. 1a,b), or bafilomycin A1, a lysosomal inhibitor (Supplemental Fig. 1a). Consistent with this result, the SQSTM1 level was significantly decreased by nPt (Fig. 1c). Similar results were obtained in HTR8/SV40neo cells, another EVT cell line (Supplemental Fig. 1b–d). Immunocytochemical analysis of MAP1LC3B also confirmed an increase of dots representing autophagosome formation in the cells subjected to nPt (Fig. 1d). The number of MAP1LC3B dots was significantly higher in the cells with nPt than in the controls regardless of the presence of E64 + P (Fig. 1e). In the presence of nPt, lysosomal inhibition by E64 + P significantly increased the number of MAP1LC3B dots, indicating activation of autophagy flux by nPt. In addition, nPt inhibited cellular proliferation in a dose-dependent manner in HchEpC1b cells (Fig. 1f). Cellular proliferation was also inhibited by nPt in HTR8/SV40neo cells, BeWo cells, a choriocarcinoma cell line, and human umbilical vein endothelial cells (HUVECs), which were used in the double cell tube formation assay (Supplemental Fig. 2a–c). Taken together, nPt, which exhibit cellular cytotoxicity, activated autophagy in the trophoblast cell lines.

### Autophagy protects against functional impairment by nPt in trophoblast cells

**Figure 2:**
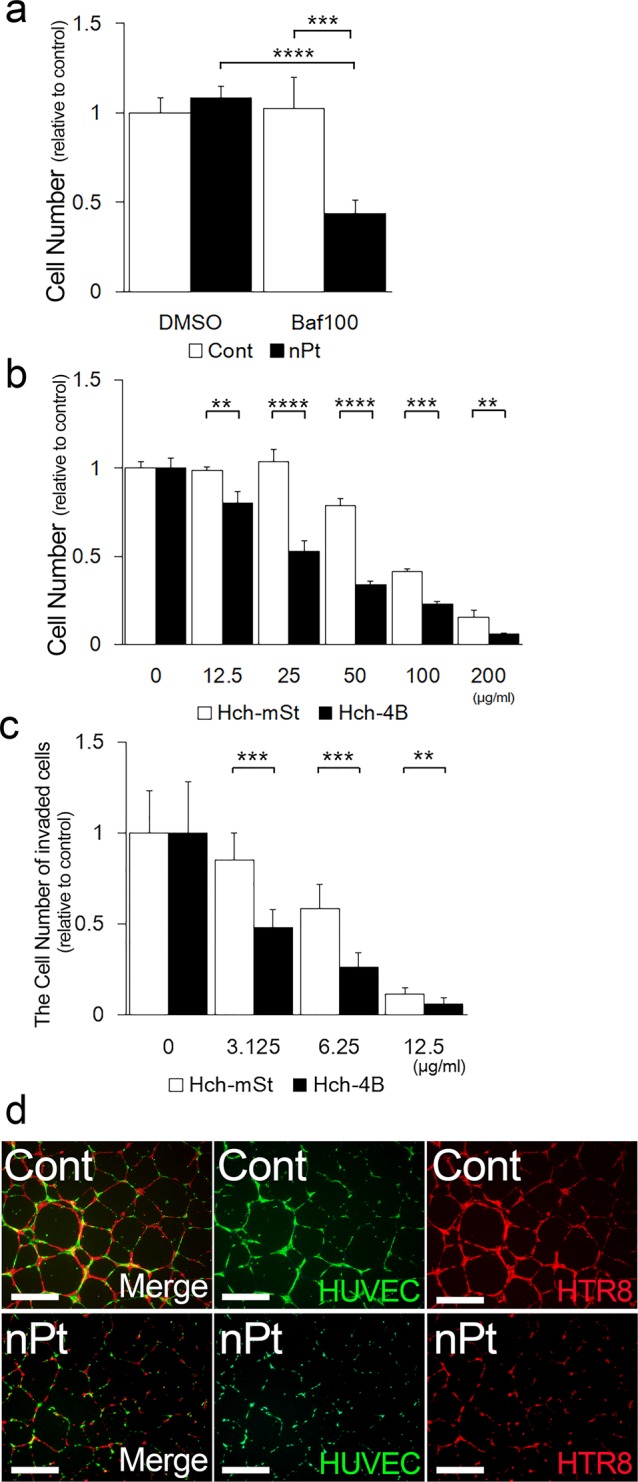
Functional impairment of EVT cells by nPt. (**a**) The cellular proliferation assay was performed using HchEpC1b cells cultured with 25 μg/ml of nPt (black bars) in the presence or absence of 100 nM bafilomycin A1 (Baf) for 24 h. (**b**) The cellular proliferation assay performed using HchEpC1b-ATG4B^C74A^ (Hch-4B, black bars) cells, an autophagy-deficient EVT cell line, or HchEpC1b-mStrawberry (Hch-mSt, white bars) cells, an autophagy-normal cell line, cultured with nPt at the indicated concentrations (μg/ml). (**c**) An *in vitro* invasion assay was performed with HchEpC1b-ATG4B^C74A^ cells (black bars) or HchEpC1b-mStrawberry cells (white bars) in the presence of nPt at the indicated concentrations (μg/ml) for 24 h. The numbers of cells in the treatment groups were normalized to that with control treatment, PBS, as one. (**d**) Tube formation assays were performed on Matrigel with HUVECs, which were labeled with green, and HTR8/SV40neo cells, which were labeled with red, in the presence of 6.25 μg/ml of nPt for 12 h. Representative images were from cultures at 12 h. Data were expressed as mean ± S.D. **p < 0.01, ***p < 0.001, ****p < 0.0001. Scale bar: 500 μm.

To further clarify the involvement of autophagy, a cellular proliferation assay was performed in the presence of bafilomycin A1, which inhibits autophagy by blocking the fusion of lysosomes with autophagosomes. Administration of 25 μg/ml of nPt inhibited the cellular proliferation of HchEpC1b cells in the presence of bafilomycin A1 but not in the presence of DMSO (Fig. 2a). The inhibition of cellular proliferation by nPt was augmented by bafilomycin A1 in a concentration-dependent manner, as compared with that without nPt (Supplemental Fig. 3a). We next evaluated the inhibitory effects of nPt in HchEpC1b-ATG4B^C74A^ cells, whose autophagy flux was blocked by the stable expression of mutant ATG4B^38^ (Supplemental Fig. 3b). The inhibitory effects of nPt on proliferation were higher in autophagy-deficient HchEpC1b-ATG4B^C74A^ cells than in autophagy-normal HchEpC1b-mStrawberry cells (Fig. 2b). We further evaluated the effects of nPt on EVT functions, such as invasion and vascular remodeling, which are required for normal placentation. As expected, invasion of HchEpC1b-mStrawberry cells with nPt was attenuated in a dose-dependent manner (Fig. 2c, white bars), but the inhibitory effects by nPt were more marked in HchEpC1b-ATG4B^C74A^ cells than in HchEpC1b-mStrawberry cells (Fig. 2c, black bars). Of note, invasion was impaired at 3.125 or 6.25 μg/ml of nPt, which had minimal inhibitory effects on cellular proliferation (Fig. 2b). An *in vitro* tube formation assay using two cell lines, HTR8/SV40neo cells and HUVECs, was performed to evaluate vascular remodeling by EVT cells^39^. We fixed the concentration of nPt at 6.25 μg/ml, which did not inhibit cellular proliferation in HTR8/SV40neo cells or HUVECs (Supplemental Fig. 2a,c). The tube-like structures, which were constructed together with these two cell lines, were impaired by 6.25 μg/ml of nPt (Fig. 2d). When using HchEpC1b cells instead of HTR8/SV40neo cells, the tube-like structure was disrupted by 3.125 μg/ml of nPt (Supplemental Fig. 3c). Poor tubulation in the assay with nPt was confirmed by evaluating the total tube area, number of segments, total tube length, and the number of total branch points (Supplemental Fig. 3d). These results suggested that basal autophagy is involved in protecting the cells from cytotoxicity as well as in sustaining functions in EVT cells.

### Accumulation of nPt in the autophagy-deficient trophoblast cells

**Figure 3:**
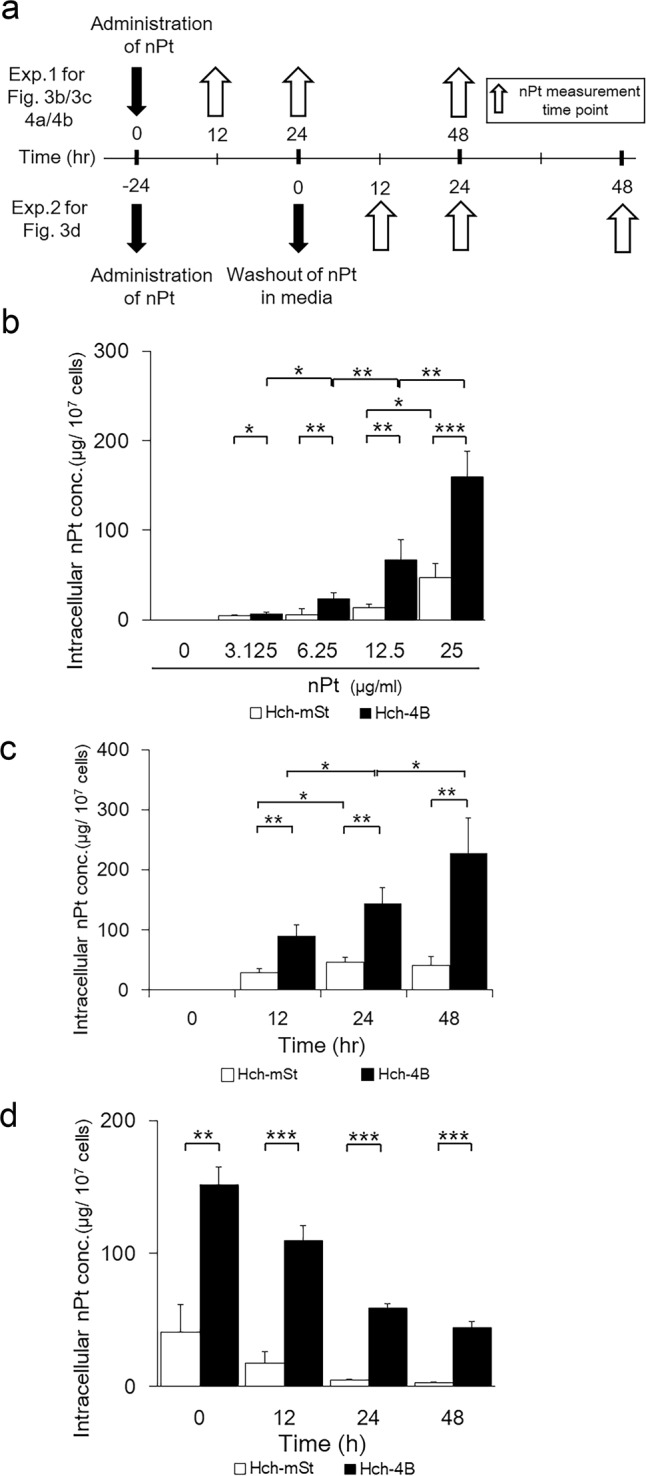
Higher accumulation of nPt in autophagy-deficient cells than in autophagy-normal cells. (**a**) The schedule of nPt administration; experiment 1 (upper) shown in (**b**,**c**), 4a,4b was to examine the uptake of nPt into the cells, and experiment 2 (lower) shown in (**d**) was to examine the retention of nPt in the cells. For experiment 2, cells cultured with nPt for 24 h were washed with PBS and fresh media was added. Then, intracellular nPt concentrations were measured at 12, 24, or 48 h. (**b**,**c**) The graph showed the intracellular nPt concentrations in the HchEpC1b-ATG4B^C74A^ cells (black bars), the autophagy-deficient cells, and HchEpC1b-mStrawberry cells (white bars), the autophagy-normal cells, cultured with nPt at the indicated concentrations (μg/ml) for 24 h (**b**) or with 25 μg/ml nPt at the indicated time (**c**). (**d**) Intracellular nPt concentrations were measured in the HchEpC1b-ATG4B^C74A^ cells (black bars) or HchEpC1b-mStrawberry cells (white bars) cultured with 25 μg/ml nPt for 24 h or at the indicated time since media was replaced. Data are expressed as the mean ± S.D. *p < 0.05, **p < 0.01, ***p < 0.001.

To examine the dynamic state of nPt in trophoblast cells, intracellular nPt concentrations were examined in HchEpC1b-ATG4B^C74A^ cells and HchEpC1b-mStrawberry cells. As shown in Fig. 3a, uptake of nPt was examined initially (exp. 1), and retention of nPt was then measured after washing out the nPt in media (exp. 2). The intracellular nPt concentrations were significantly higher in HchEpC1b-ATG4B^C74A^ cells than in HchEpC1b-mStrawberry cells, and the concentrations were dose-dependently increased in both cell lines (Fig. 3b). The accumulation of nPt was also observed in ATG5 knockout mouse embryonic fibroblasts (Supplemental Fig. 4a). The time-dependency was also confirmed in HchEpC1b-ATG4B^C74A^ cells but not in HchEpC1b-mStrawberry cells (Fig. 3c), suggesting that autophagy deficiency led to the nPt accumulation in trophoblast cells. Next, to clarify the intake mechanism for nPt into the trophoblast cell lines, endocytosis was blocked by several inhibitors^40^; chlorpromazine (CPZ) for clathrin, methyl-β-cyclodextrin (MβCD) for caveolae, or amiloride (ALR) for micropinocytosis. These inhibitors, however, did not inhibit the intake of nPt into HchEpC1b-ATG4B^C74A^ cells or -mStrawberry cells (Supplemental Fig. 4b), and the intake of nPt was instead increased in the presence of CPZ or MβCD in HchEpC1b-ATG4B^C74A^ cells. Additionally, these inhibitors, which did not inhibit cell proliferation without nPt, did not rescue the reduced cell number by nPt in either cell line (Supplemental Fig. 4c,d). To further examine the retention of nPt, the intracellular nPt concentration was also examined following exclusion of nPt from the media (Fig. 3a, exp. 2). As a result, the intracellular nPt concentration was higher in HchEpC1b-ATG4B^C74A^ cells even at 48 h after washing (Fig. 3d). The amount of nPt retained in the cells decreased in a time-dependent manner in both cell lines, but rates were significantly higher in HchEpC1b-ATG4B^C74A^ cells until 48 h (37.5 ± 8.4% vs 72.2 ± 11.4% at 12 h, 10.3 ± 0.6% vs 39.0 ± 2.9% at 24 h, 5.5 ± 0.7% vs 29.1 ± 4.9% at 48 h, Supplemental Fig. 4e). Taken together, autophagy deficiency may be one cause of the accumulation of nPt, suggesting failure of nPt evacuation in the autophagy-deficient trophoblast cells.

### nPt in the autophagosome

**Figure 4:**
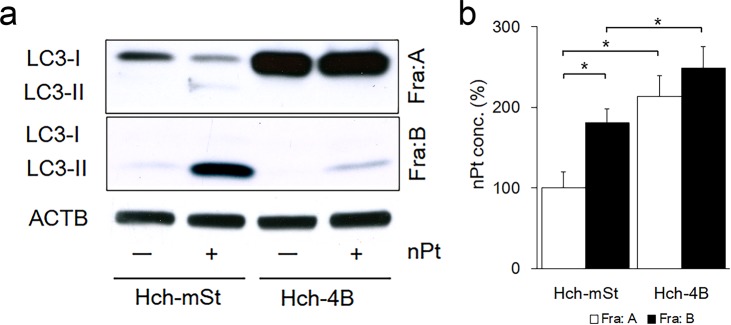
Sequestration of nPt in the autophagosome-rich fraction. (**a**) Cell lysates of HchEpC1b-mStrawberry (Hch-mSt) cells or HchEpC1b-ATG4B^C74A^ (Hch-4B) cells cultured with 25 μg/ml of nPt (+) or (−), PBS, were separated into fraction A (Fra: A), which mainly contained the cytoplasm, and fraction B (Fra: B), which mainly contained autophagosomes. (**b**) The graph showed the intracellular nPt concentrations in fraction A and B of HchEpC1b-mStrawberry cells and HchEpC1b-ATG4B^C74A^ cells cultured with 25 μg/ml of nPt for 24 h. The nPt concentrations were normalized to that in fraction A of HchEpC1b-mStrawberry cells as 100%. Data were expressed as the mean ± S.D. *p < 0.05.

To further investigate the correlation between nPt and autophagy in detail, cell lysates were separated into the MAP1LC3B-I fraction, which mainly contains the cytoplasmic fraction, referred to as fraction A, or the MAP1LC3B-II fraction, which is abundant in autophagosomes and referred to as fraction B. MAP1LC3B-II proteins are only present in autophagosomes. As shown in Fig. 4a, the main MAP1LC3B-II proteins were localized in fraction B, suggesting that fraction B is an autophagosome-rich fraction. The nPt concentration in the autophagosome-rich fraction B was significantly higher than that in fraction A in the HchEpC1b-mStrawberry cells, but no significant difference was noted between fraction A and B in the autophagy-deficient HchEpC1b-ATG4B^C74A^ cells (Fig. 4b). These results speculated that nPt were trapped by autophagosomes in the autophagy-normal trophoblast cells.

### DNA damage by nPt in autophagy-deficient cells

**Figure 5:**
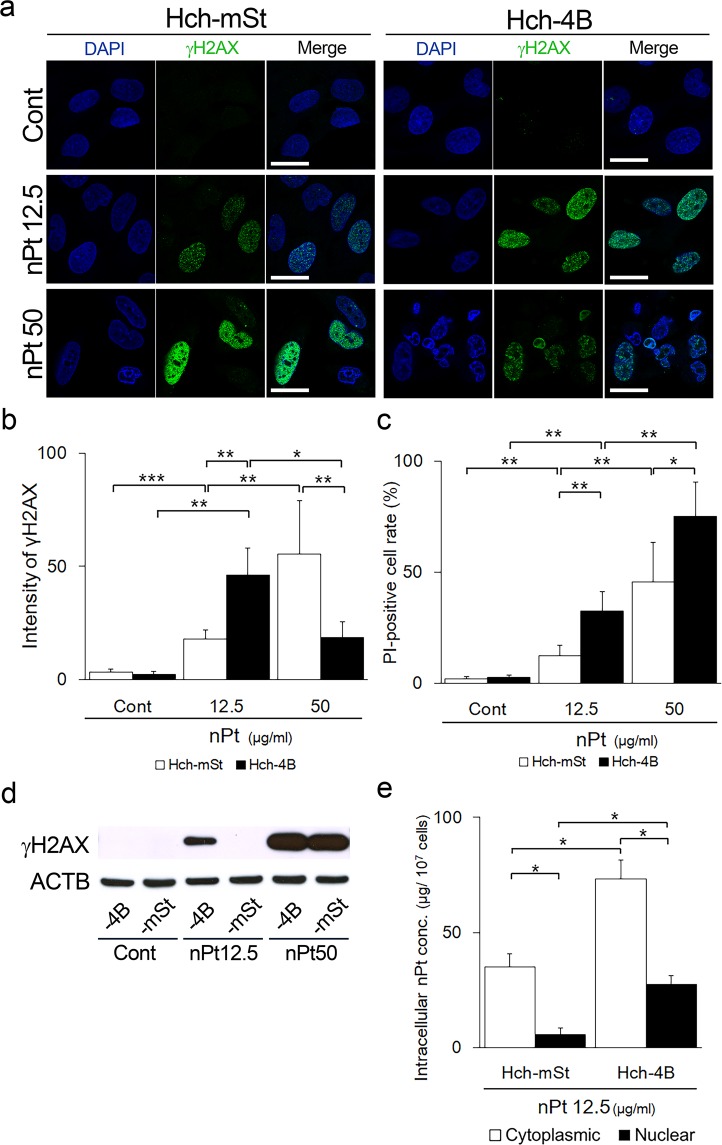
Autophagy protected cells from nPt-mediated DNA damage. (**a**) Representative panels showed the nuclei staining (DAPI, blue), single staining of anti-phospho-γH2AX (γH2AX, green), and the merged images of the HchEpC1b-mStrawberry (Hch-mSt) cells or HchEpC1b-ATG4B^C74A^ (Hch-4B) cells cultured with 12.5 μg/ml or 50 μg/ml of nPt for 24 h. (**b**) The average intensity of anti-γH2AX staining in each cell was shown in the graph. White bars indicated the Hch-mSt cells and black bars indicated Hch-4B cells. (**c**) The proportion of propidium iodide (PI)-positive Hch-mSt cells (white bars) or Hch-4B cells (black bars), which were cultured with 12.5 μg/ml or 50 μg/ml of nPt for 24 h, was shown. (**d**) Western blots of Hch-mSt or -4B cells, which were cultured with 12.5 or 50 μg/ml of nPt for 24 h, were as follows: γH2AX, and ACTB. (**e**) The graph showed the nPt concentrations in cytoplasmic (white bars) or nuclear (black bars) fraction in Hch-mSt or -4B cells treated with 12.5 μg/ml of nPt for 24 h. Data are expressed as the mean ± S.D. *p < 0.05, **p < 0.01, ***p < 0.001. Scale bars: 15 μm.

Platinum-containing medications, such as in chemotherapeutic agents, are known to induce DNA damage by cross-linking DNA. We, therefore, examined the effects of uncaptured nPt on DNA damage in the autophagy-deficient HchEpC1b-ATG4B^C74A^ cells. DNA damage, which was estimated by phosphorylated γH2AX (p-γH2AX) staining, induced by 12.5 μg/ml of nPt was more marked in the HchEpC1b-ATG4B^C74A^ cells than that in the HchEpC1b-mStrawberry cells (Fig. 5a). When administered 50 μg/ml of nPt, the intensity of p-γH2AX staining was strong in HchEpC1b-mStrawberry cells; however, shrunken nuclei and p-γH2AX-positive cells were observed more in HchEpC1b-ATG4B^C74A^ cells (Fig. 5a). Indeed, the intensity of p-γH2AX staining was the highest with 12.5 μg/ml of nPt in HchEpC1b-ATG4B^C74A^ cells and with 50 μg/ml of nPt in HchEpC1b-mStrawberry cells (Fig. 5b). Similar results were obtained for the amount of p-γH2AX-positive cells (Supplemental Fig. 5a). On the other hand, the amount of propidium iodide-positive cells, which indicates dead cells, was the highest with 50 μg/ml of nPt in HchEpC1b-ATG4B^C74A^ cells (Fig. 5c). To further confirm the DNA damage by nPt, immunoblotting of p-γH2AX and the comet assay were performed. p-γH2AX expression was observed in the HchEpC1b-ATG4B^C74A^ cells but not in HchEpC1b-mStrawberry cells with 12.5 μg/ml of nPt (Fig. 5d). The comet tail length was also significantly longer in HchEpC1b-ATG4B^C74A^ cells than in HchEpC1b-mStrawberry cells when treated with 12.5 μg/ml of nPt (Supplemental Fig. 5b). Lastly, the nPt concentration was compared between the nuclear and cytoplasmic fractions in the cells with 12.5 μg/ml of nPt. The amount of nPt in the nuclear fraction was significantly higher in HchEpC1b-ATG4B^C74A^ cells than in HchEpC1b-mStrawberry cells (Fig. 5e). The proportion of nPt in the nuclear fraction to the total was 27.3% in HchEpC1b-ATG4B^C74A^ cells and 14.0% in HchEpC1b-mStrawberry cells. These results demonstrated that more nPt accumulated in autophagy-deficient cells, making them more vulnerable to DNA damage induced by nPt without autophagy.

## Discussion

**Figure 6:**
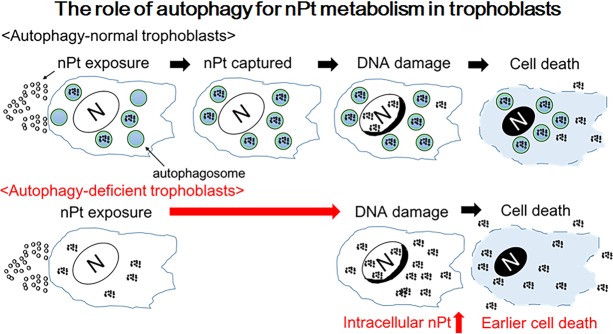
Hypothesis: the role of autophagy in nPt metabolism in trophoblast cells. When nPt intrude into trophoblast cells, they are initially captured by autophagosomes. However, excessive nPt that escape from autophagosomes may accumulate in nuclei and damage DNA, resulting in cell death. On the other hand, nPt easily accumulate in nuclei in autophagy-deficient cells because of the lack of autophagosomes. As a result, autophagy-deficient cells may be sensitive to the cytotoxicity of nPt.

We found that nPt activated autophagy flux, which was confirmed by administering lysosomal inhibitors in two EVT cell lines. In addition, nPt inhibited EVT functions and cellular proliferation. Lower concentrations of nPt affected EVT functions more than proliferation, whereas higher concentrations of nPt induced cell death, resulting in the inhibition of proliferation. Moreover, autophagy-deficient cells were more susceptible to the inhibitory effects of nPt than autophagy-normal cells. We observed that nPt accumulated more in the autophagosome-rich fractions in the autophagy-normal EVT cell line. Lastly, more nPt accumulated in the nuclei of autophagy-deficient cells, resulting in greater DNA damage induced by nPt at a lower concentration. Our hypothesis is illustrated in Fig. 6. When autophagy-normal cells are exposed to nPt, excessive nPt, some of which are initially captured by autophagosomes, may accumulate in nuclei, resulting in DNA damage-induced cell death. However, nPt easily accumulate in nuclei in autophagy-deficient cells because of the lack of autophagosomes, which enables nPt to enter the nuclei of autophagy-deficient cells. Thus, segmentation of nPt by autophagosomes reduces the cytotoxicity of nPt (as shown in Figs 2b and 4b) because nPt exhibited greater cytotoxicity in the autophagy-deficient cell line in which the ATG4B^C74A^ mutant blocks the closure of autophagosomes^38^. On the other hand, CPZ, which inhibits clathrin, increased the intracellular nPt concentrations in both autophagy-normal and –deficient cells (Supplemental Fig. 4b). This suggests that nPt intake depends on other endocytic mechanisms in trophoblasts. Further studies are needed to clarify this.

In pregnant mice administered silica nanoparticles, which were found distributed in the liver and placenta, the proportion of spongiotrophoblast layer area to the total placental area was approximately 50% smaller than that in the control^7^. In humans, placental growth is partially dependent on EVT functions of invasion and vascular remodeling during early pregnancy^41^. As EVT functions are supported by autophagy^41,42^, impairment of autophagy was reported to be involved in the inhibition of placental growth and pregnancy complications such as gestational hypertension or premature delivery^41,43,44^. Consistent with this, our study demonstrated that autophagy protected against nPt-mediated trophoblast injury. Thus, nPt may exacerbate placenta-mediated pregnancy complications, such as preeclampsia or FGR, especially in autophagy-impaired placentas. Indeed, placenta-specific autophagy-deficient mice exhibited poor placentation with impairment of invasion and vascular remodeling, resulting in gestational hypertension^44^. Furthermore, some nanoparticles are related to placental inflammation via the inflammasome^16,45^, which worsens preeclampsia. Although there are structural differences between human and mouse placentas, smaller nanoparticles that escape from autophagosomes can elicit cell death in trophoblast cells, resulting in placental inflammation in humans. Thus, measurement of nanoparticle concentrations in the placenta may be a future focus for nano-safety research.

The inhibition of autophagy by nPt was reported in a human lymphoma cell line^46^, but the precise mechanism by which pretreated nPt inhibited autophagy was not described. Autophagic responses vary with different sizes or physicochemical properties of nanoparticles^47^, and the degree of autophagic activity or responses induced by even the same stimulus are also different in each organ^48^. Thus, the autophagic response to nPt may differ in each cell line. This study, however, revealed that autophagy was activated by nPt in two EVT cell lines. For the functional assays of EVT cells, a lower concentration of nPt (e.g. 6.25 μg/ml), which had little effect on proliferation, inhibited invasion and disrupted the tube formation of EVT cells. This means that the functions of EVT cells rather than cellular proliferation are more sensitive to nPt cytotoxicity. The causes are not clear, but accumulation of nPt in nuclei may have led to this result. From a clinical point of view, a lower concentration of nPt may affect placentation in early pregnancy. Therefore, the use of nPt for pregnant women is of concern. Silica nanoparticles were reported to impair endothelial function via autophagy activation in HUVECs^26^, and similar results were obtained for co-cultured HTR8/SV40neo cells and HUVECs subjected to nPt. Thus, nPt may be also lead to poor placentation through dysfunction in both EVT and endothelial cells.

Regarding the nPt-induced autophagy, xenophagy, which is selective autophagy for eliminating pathogens, may recognize nPt as small pathogens^49^. The intracellular dynamics of nPt have not been elucidated because we cannot detect these nanoparticles in cells even by electron microscopy. Therefore, we estimated the intracellular distribution of nPt by dividing the autophagosome-rich, fraction B, and non-rich fractions, fraction A. We speculated that more nPt were located in autophagosomes, but it is possible that nPt are located in other types of vesicles such as endocytic vesicles. To clarify the significance of autophagy for nPt, we compared the dynamic state of nPt between the autophagy-normal and –deficient cells. The amount of nPt was time-dependently increased in the autophagy-deficient cells but not in the autophagy-normal cells (Fig. 3c). On the other hand, the proportion of retained nPt was significantly lower in the autophagy-normal cells than in the autophagy-deficient cells (Supplemental Fig. 4e). Additionally, nPt may be trapped by autophagosomes. Based on these results, we hypothesized that the selected autophagy pathway was secretion rather than degradation because nPt were unable to be degraded by autophagic machinery. Autophagic vesicles were reported to release carbon nanotubes into the extracellular space to reduce the cytotoxicity in HUVECs, for unconventional secretion of IL-1β, and to release the Epstein Barr virus^50–52^. To confirm this possibility, we measured the concentrations of nPt released into the media. They were much lower than those of intracellular nPt (data not shown) Furthermore, we were unable to distinguish nPt in the media from those actively released by autophagy in live cells or passively leaked from dead cells. Therefore, autophagy may function in the extracellular release of nPt, but further examinations, such as microscopic observation of the intracellular localization of nPt, are required to confirm our hypothesis.

This study elucidated the physiological importance of nPt in trophoblast cells during early placentation. As some nanoparticles can accumulate in the placenta or liver in pregnant mothers, even low concentrations of nPt must be monitored for pregnant women. Use of nPt may inhibit placental growth or exacerbate placenta-mediated pregnancy complications. *In vivo* studies are necessary to confirm this hypothesis. On the other hand, this study also suggested that nPt are metabolized by autophagy machinery (Fig. 6). Biocompatible surface coating of nanoceria is known to activate autophagy clearance mediated by transcriptional factor EB^28^. Although surface modifications, which dominate the chemical reaction, of nPt can reduce the negative effects on trophoblast cells, smaller sized nanoparticles should be avoided for pregnant women.

## Methods

### Reagents

Platinum nanoparticles (nPt) with a nominal mean diameter of less than 1 nm, which was confirmed with a Zetasizer Nano-ZS (Nano platinum water based solution, Malvern Instruments, Malvern, UK), were from Polytech & Net GmbH (Schwalbach, Germany). The particles were not physically modified, and stocked in aqueous solution at 5 mg/ml (Supplemental Fig. 6). The particles were sonicated for 5 min and vortexed for 1 min prior to use. The following mouse monoclonal antibodies (Ab) were used: anti- α-tubulin (T8203, Sigma-Aldrich, MA, USA), anti-p62/SQSTM1 (M162-3, MBL, Nagoya, Japan), anti-phospho Histone 2AX (Ser139) (05–636, Merck Millipore, Burlington, MA, USA), anti-Histone 3 (9715, Cell Signaling Technology (CST), MA, USA), and normal mouse IgG (sc-2025, Santa Cruz Biotechnology, Dallas, TX, USA). The rabbit polyclonal Ab anti-MAP1LC3B (PM036, MBL) and normal rabbit IgG (sc-2027, Santa Cruz Biotechnology) were used. The following secondary Ab were used: anti-mouse IgG-HRP conjugate (7076, CST), anti-rabbit IgG-HRP conjugate (7074, CST), Alexa Fluor 488 donkey anti-rabbit IgG (A-21206, Thermo Fisher Scientific, Waltham, MA, USA), and Alexa Fluor 488 donkey anti-mouse IgG (A-21202, Thermo Fisher Scientific). Chemical inhibitors used were: bafilomycin A1 (11038, Cayman Chemical, Ann Arbor, USA), a V-ATPase inhibitor, and, E64d (4321-v, Peptide institute Inc., Osaka, Japan) and pepstatin A (4397, Peptide institute Inc.), protease inhibitors, chlorpromazine (CPZ, C2481, Tokyo Chemical Industry Co. Ltd., Tokyo, Japan), an inhibitor of clathrin, methyl-beta-cyclodextrin (MβCD, C0777, Tokyo Chemical Industry Co. Ltd.) an inhibitor of caveolae, or amiloride (ALR, A259, Tokyo Chemical Industry Co. Ltd), an inhibitor of macropinocytosis.

### Cell culture and chemical treatments

Two EVT cell lines, HTR8/SV40neo and HchEpC1b, and ATG5 knockout mouse embryonic fibroblasts were used in this study. HTR8/SV40neo cells were immortalized by introducing the simian virus 40 large T antigen^53^, and HchEpC1b cells were immortalized by introducing the type 16 human papillomaviruses E6 and E7 combined with human telomerase reverse transcriptase^54^. Furthermore, autophagy-deficient cell lines, HTR8-ATG4B^C74A^ and HchEpC1b-ATG4B^C74A^, and control cell lines, HTR8-mStrawberry and HchEpC1b-mStrawberry, which we previously established, were also used^41^. These cells were cultured in RPMI1640 medium (11875, GIBCO, MA, USA) supplemented with 10% FBS, 100 U/ml of penicillin, and 100 µg/ml of streptomycin (15140, GIBCO) at 37 °C in a 5% CO_2_ atmosphere. For cell cultures with nanoparticles, cells were grown in media for 24 h prior to treatment. Then, the culture medium was exchanged for fresh medium with each nanoparticle type, and cells were cultured in the media for 12 to 48 h. The particles were sonicated for 5 min just prior to adding them to the media. For cultures with chemical inhibitors, the cells were treated with protease inhibitors, E64d (10 ng/ml) and pepstatin A (10 ng/ml) for 2 h to inhibit autophagy flux on Western blotting. In addition, bafilomycin A1 (range from 0 to 200 nM), an inhibitor of V-ATPase, was also used for 24 h to inhibit autophagy flux.

### Western blotting

Western blotting was carried out as described previously^55^. In brief, cells were washed with cold PBS 3 times to completely remove nanoparticles, and then harvested and lysed in RIPA buffer (9806, CST) containing protease inhibitor cocktail (P8340, Sigma-Aldrich) and 1% phosphatase inhibitor (P5726, Sigma-Aldrich). The lysates were centrifuged at 14000 rpm for 30 min at 4 °C. The protein concentration was measured using a Bradford protein assay kit (T9310A, Takara, Shiga, Japan). Protein samples were then mixed with a 2 × Laemmli sample buffer (161–0737, Bio-Rad, CA, USA) and heated at 95 °C for 5 min. Equal amounts of protein were then applied to 5–20% SuperSep® Ace precast gels (Wako Pure Chemical Industries Ltd., Osaka, Japan) and transferred to Immun-Blot PVDF membranes (1620174, Bio-rad). The membranes were blocked with 5% skim milk buffer for 1 h at room temperature, and then incubated overnight at 4 °C with the following ab: anti-ACTB (1:5000), anti-MAP1LC3B (1:1000), and anti-p62/SQSTM1 (1:2000). Lastly, they were incubated for 1 h with HRP-conjugated anti-mouse or anti-rabbit ab and visualized with an enhanced chemiluminescence detection system (32106, PIERCE, Rockford, IL, USA).

### Separation of two fractions, MAP1LC3B-I-rich and MAP1LC3B-II-rich fractions, or nuclear and cytoplasmic

Cells were washed with cold PBS 3 times to completely remove nanoparticles, and then harvested and lysed in 10 mM Tris-HCl (312-90061, Nippon Gene, Tokyo, Japan) with protease inhibitor cocktail and 1% phosphatase inhibitor. The supernatant was obtained from the lysates, which were centrifuged at 14000 rpm for 30 min at 4 °C, and stocked as fraction A, the MAP1LC3B-I-rich fraction. The pellet at the bottom was washed with cold PBS 3 times, and PBS was removed. The pellet was further lysed in RIPA buffer containing protease inhibitor cocktail, 1% phosphatase inhibitor, and 0.5% Sodium dodecyl sulfate (L3771, Sigma-Aldrich). The lysates were centrifuged at 14000 rpm for 30 min at 4 °C, and the supernatants were stocked as fraction B, the MAP1LC3B-II-rich fraction. The remaining pellet was discarded. To separate the cytoplasmic and nuclear fractions, the NE-PER Nuclear and Cytoplasmic fraction kit was used following the manufacturer’s instructions (78833, Thermo Fisher Scientific). The separated fractions were confirmed by Western blotting of ACTB or H3F3B (Supplemental Fig. 6b).

### Immunocytochemistry

Immunocytochemistry was carried out as described previously^56^. Cells were fixed with 4% paraformaldehyde/PBS for 15 min, permeabilized, and blocked with PBS containing 3% bovine serum albumin (A2153, Sigma-Aldrich) with 0.1% Triton X-100 (T9284, Sigma-Aldrich). Subsequently, cells were labeled with the primary ab, anti-MAP1LC3B (1/500) or anti-pH2AX (1/250). For negative controls, the primary ab was replaced with normal rabbit IgG or normal mouse IgG. After washing out the primary ab, cells were secondarily stained with Alexa Fluor 488 donkey anti-rabbit IgG (1/1000) or Alexa Fluor 488 donkey anti-mouse IgG (1/1000), followed with Hoechst33342 nuclear staining for 10 min. Cells were observed using a confocal microscope (LSM700, Carl Zeiss, Oberkochen, Germany).

### Invasion assay

The conventional invasion assay was performed using a BD BioCoat Growth Factor Reduced Matrigel Invasion Chamber (354483, BD Biosciences, NJ, USA) as described previously^42^. In brief, cells, which were plated on the upper insert at 1 × 10^5^/well with or without nanoparticles, were incubated for 24 h. The invaded cells on the under surface of the membrane were fixed after removing the cells on the upper surface of the membrane in each insert. The number of invaded cells, which were stained with 0.05% Toluidine Blue Solution (206-14555, Wako), was manually counted by microscopy.

### Double cell tube formation assays

This assay was reported previously^39^. In brief, for the double cell tube formation assay, EVT cells (1 × 10^5^/ml), which were labeled with cell tracker red CMTMR (5-(and-6)-(((4-chloromethyl)benzoyl)amino) tetramethylrhodamine, C-2927, Molecular Probes), and HUVECs (1 × 10^5^/ml), which were labeled with cell tracker green CMFDA (5-chloromethylfluorescein diacetate, C-2925, Molecular Probes, MA, USA), were mixed and cultured on plates covered with thick Matrigel (356234, BD) in the presence or absence of nanoparticles at 37 °C for 24 h. The number of tube-like structures per image, and their total area, number of segments, total length, and number of branching points (more than 2) were quantified and averaged for five independent visual fields using Image J (http://rsbweb.nih.gov/ij/).

### Cell proliferation assay

Two methods, manual counting or the cell proliferation reagent WST-1 (Roche, 5015944), were used for estimating cell proliferation. In brief, WST-1 reagent was added to the media with nanoparticles in which cells were cultured. Two hours later, the absorbance was measured using a microplate reader (1681135JA, iMark microplate reader, Bio-rad) at a wavelength of 450 nm. The reference wavelength was 650 nm.

### Dead cell detection

The number of cells cultured with nanoparticles that were stained or unstained with 0. 4% Trypan Blue Solution (15250061, Thermo Fisher) were counted. Then, the proportion of stained cells among all cells was calculated as dead cells. Fixed cells were also stained with propidium iodide solution (P4864, 1 mg/ml Sigma-Aldrich), which was used at 2 μg/ml diluted in a staining buffer (100 mM Tris, 150 mM NaCl, 1 mM CaCl2, 0.5 mM MgCl2, 0.1% Nonidet P-40). The positive cells were counted as dead cells.

### Quantitative analysis of GFP-LC3 puncta

This assay was previously reported^41^. For the quantitative analysis of MAP1LC3B, the cells were stained with anti-MAP1LC3B. The cells were pretreated with lysosomal protease inhibitors, E64d (10 ng/ml) and pepstatin A (10 ng/ml), for 2 h before fixation. The number of MAP1LC3B puncta in a single cell was estimated by manual counting in at least thirty cells using a confocal microscope.

### Measurement of nPt concentrations

Cellular samples, which were washed with cold PBS 3 times followed by treatment with trypsin-EDTA (T4049, Sigma-Aldrich) for 5 min, were counted, and all samples were simultaneously lysed with the same amount of RIPA buffer. Media samples were collected, and the volume of the samples was measured. Subsequently, media samples were collected, and the volume of the samples was measured. The concentration of nPt in the samples was measured by inductively coupled plasma mass spectrometry (ICP-MS) with an Agilent 7700 Series instrument (Agilent Technologies, Tokyo, Japan). The instrument was operated with an RF power of 1.5 kW and a carrier gas flow rate of 1.05 L/min Ar. The following isotopes were measured: 195Pt and 205Tl. Prior to ICP-MS analysis, sample aliquots were spiked with internal standards (205Tl) at a concentration of 2 ng/mL. Element concentrations were measured by external 6-to-11-point-calibration with internal standard correction. For the control samples, cells cultured without nPt or media without nPt were used. To calculate the nPt concentrations, the obtained concentrations, which measured all platinum element, were divided by the cell number for cellular samples or by media volume for the samples. The concentrations are presented in μg/ml per 1 × 10^7^ cells for cellular samples or in μg/ml for media samples.

### Comet assay

DNA damage was assessed by OxiSelect Comet Assay kit according to the manufacture’s instruction (STA-351, Cell Biolabs, Inc., San Diego, CA, USA). Electrophoresis was performed in the alkaline solution. The data was obtained from at least 50 cell in each treated groups.

### Statistical analysis

Results are presented as the mean ± S.D., and Kruskal-Wallis and Mann-Whitney tests were used to compare the differences between groups. Values of *p* lower than 0.05 were considered significant.

## Supplementary information

Supplemental figures
