# Supplementary material for: Autophagy is a new protective mechanism against the cytotoxicity of platinum nanoparticles in human trophoblasts
Source: Sci Rep. 2019 Apr 2;9:5478. doi: 10.1038/s41598-019-41927-2 (PMC6445294; doi:10.1038/s41598-019-41927-2)

Supplemental figures

Autophagy is a new protective mechanism against the cytotoxicity of platinum nanoparticles in human trophoblasts

Akitoshi Nakashima[1], Kazuma Higashisaka[2,3], Tae Kusabiraki[1], Aiko Aoki[1], Akemi Ushijima[1],Yosuke Ono[1], Sayaka Tsuda[1], Tomoko Shima[1], Osamu Yoshino[1], Kazuya Nagano[2], Yasuo Yoshioka[2,4,5], Yasuo Tsutsumi[2,6], Shigeru Saito[1*]

[1] Department of Obstetrics and Gynecology, University of Toyama, 2630, Sugitani, Toyama 930-0194, Japan.

[2] Laboratory of Toxicology and Safety Science, Graduate School of Pharmaceutical Sciences, Osaka University, 1-6 Yamadaoka, Suita, Osaka 565-0871, Japan.

[3] Department of Legal Medicine, Graduate School of Medicine, Osaka University, 2-2 Yamadaoka, Suita, Osaka 565-0871, Japan.

[4] Vaccine Creation Project, BIKEN Innovative Vaccine Research Alliance Laboratories, Research Institute for Microbial Diseases, Osaka University, 3-1 Yamadaoka, Suita, Osaka, 565-0871, Japan.

[5] BIKEN Center for Innovative Vaccine Research and Development, The Research Foundation for Microbial Diseases of Osaka University, 3-1 Yamadaoka, Suita, Osaka, 565-0871, Japan.

[6] The Center for Advanced Medical Engineering and Informatics, Osaka University, 1-6, Yamadaoka, Suita, Osaka 565-0871, Japan.

*Correspondence to:

Shigeru Saito, email: s30saito@med.u-toyama.ac.jp, address: 2630 Sugitani, Toyama, 930-0194, Japan,

Supplemental Figure1

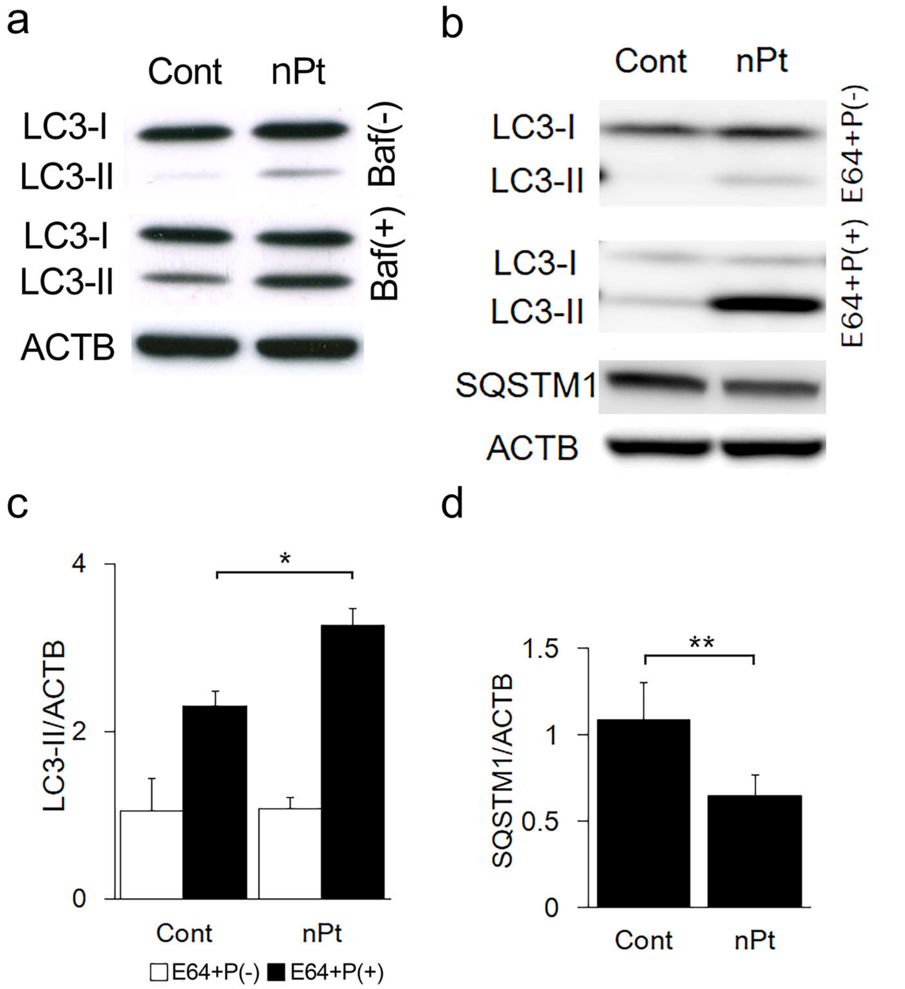

Supplemental Figure 1 Autophagy activation by nPt.

(a) Western blots of HchEpC1b cells, which were cultured with 25 µg/ml of nPt for 24 h with or without 10nM of bafilomycin A1 (Baf) for 2 h at the end of culture, were as follows: MAP1LC3B (LC3), SQSTM1, and ACTB. (b) Western blots of HTR8/SV40neo cells, which were cultured with 12.5 µg/ml of nPt for 24 h with or without E64d (E64) and pepstatin A (P) for 2 h at the end of culture, are as follows: MAP1LC3B (LC3), SQSTM1, and ACTB. The graphs showed the expression levels of MAP1LC3B-II (c) or SQSTM1 (d) in the HTR8/SV40neo cells cultured with nPt shown in (b). White bars indicate cells without E64d and pepstatin A treatment, and black bars indicate cells with E64d and pepstatin treatment. The expression was normalized to the ACTB level. *; $p < 0.05$, **; $p < 0.01$.

Supplemental figure 2

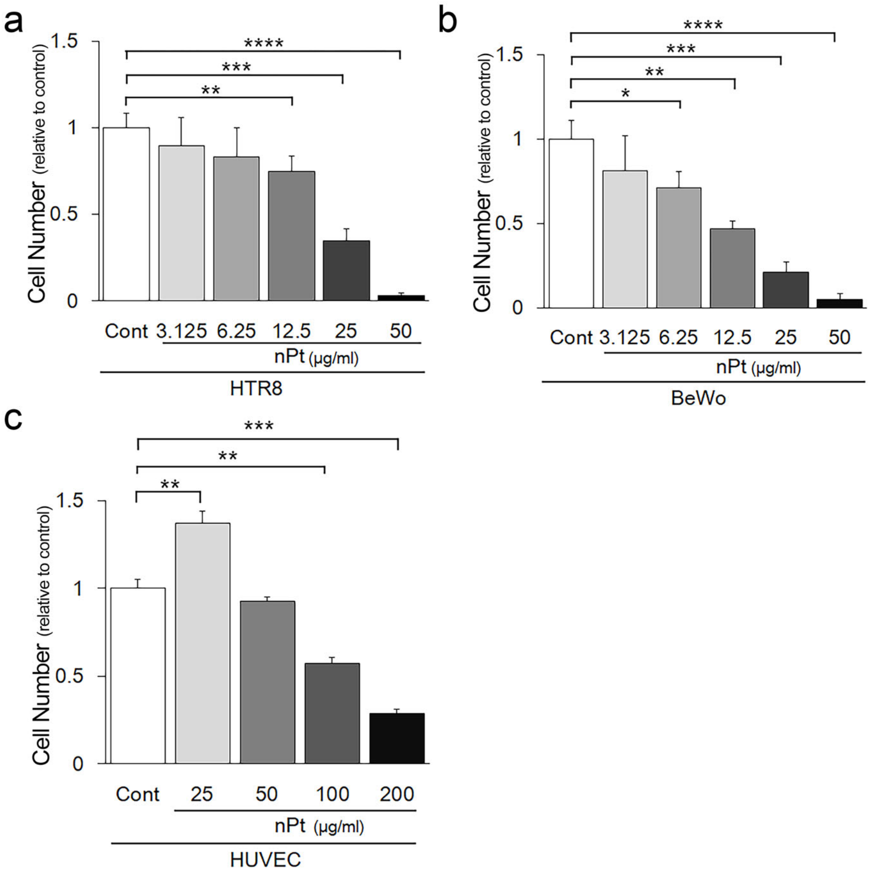

Supplemental Figure 2 Inhibition of cellular proliferation by nPt in several cell lines. Cellular proliferation of HTR8/SV40neo cells (a), BeWo cells (b), a choriocarcinoma cell line, or human umbilical vein endothelial cells (HUVECs, c) in the presence of nPt at the indicated concentrations for 24 h. For cellular proliferation assays, the number of cells in the treatment groups was normalized to that of control treatment, PBS, as one. Data are expressed as the mean ± S.D. from at least three independent experiments. *; $p < 0.05$, **; $p < 0.01$, ***; $p < 0.001$, ****; $p < 0.0001$.

Supplemental figure 3

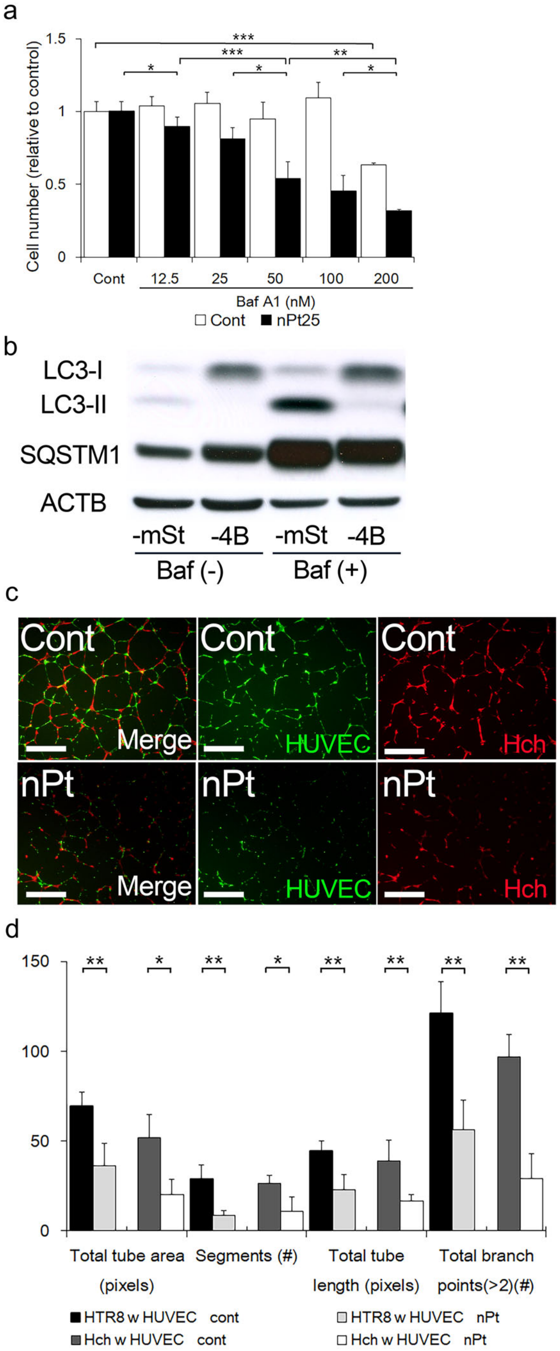

Supplemental Figure 3 Autophagy inhibition augmented nPt-mediated inhibition of cellular proliferation or tabulation in HchEpC1b cells

(a) The cellular proliferation assay was performed using HchEpC1b cells treated with or without bafilomycin A1 (Baf) at the indicated concentrations (nM) in the presence (black bars) or absence (white bars) of 25 µg/ml of nPt for 24 h. (b) Western blots of HchEpC1b-mStrawberry cells (-mSt, autophagy-normal) or HchEpC1b-$ATG4B^{C74A}$ cells (-4B, autophagy-deficient), which were cultured under serum free media for 24 h with or without 10nM of bafilomycin A1 (Baf) for 2 h at the end of culture, were as follows: MAP1LC3B (LC3), SQSTM1, and ACTB. (c) Tube formation assays were performed on Matrigel with HUVECs, which were labeled with green, and HchEpC1b cells (Hch), which were labeled with red, in the presence of 3.125 µg/ml of nPt for 12 h. (d) The tube formation was analyzed by the area, the number of segments, the total tube length, or the number of branch points (>2), in figure 2d, or supplemental figure 3c. The bars indicated the results of HTR8 with HUVEC treated with control (black) or nPt 6.25 µg/ml (light gray), or HchEpC1b with HUVEC treated with control (dark gray) or nPt 3.125 µg/ml (white). *; $p < 0.05$, **; $p < 0.01$, ***; $p < 0.001$. Scale bar: 500 µm.

Supplemental figure 4

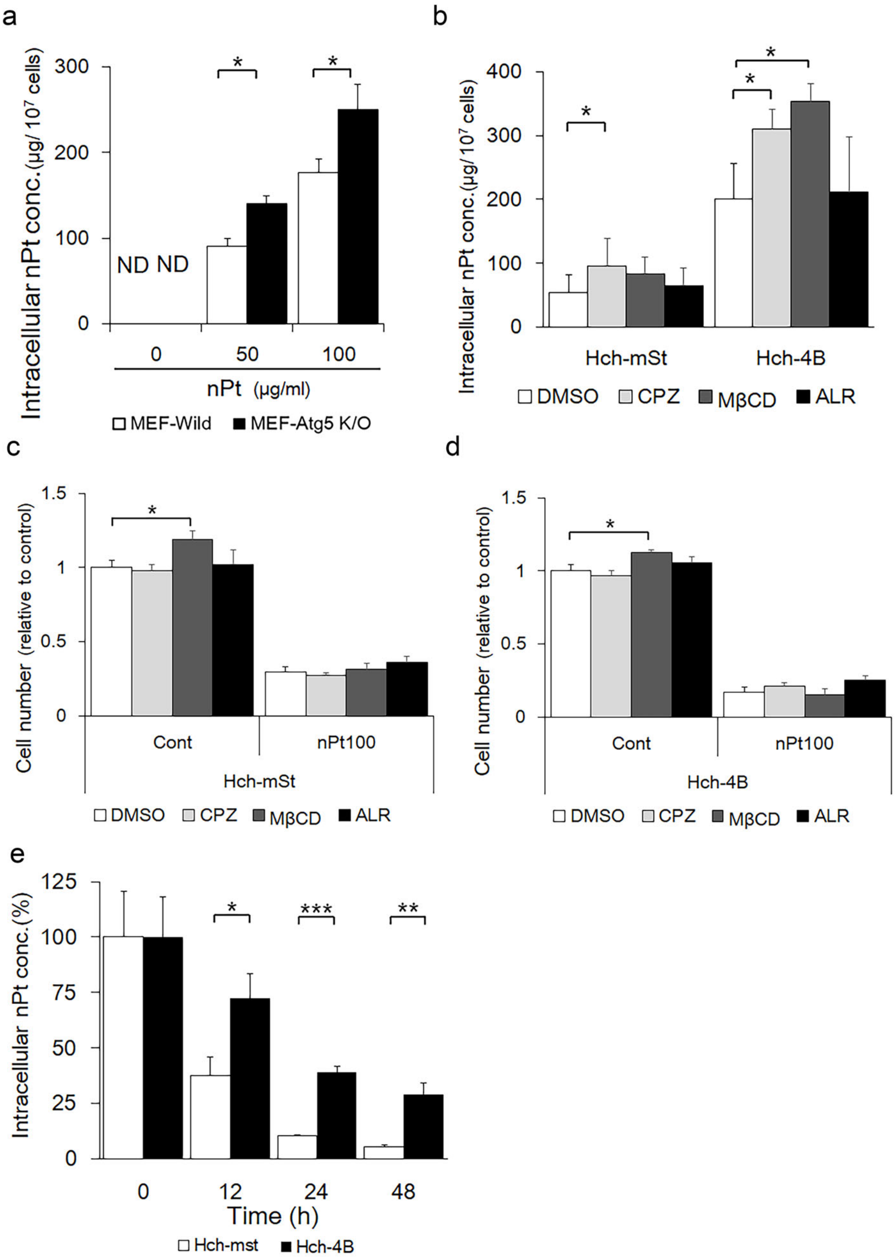

Supplemental Figure 4 nPt accumulation in autophagy-deficient cells.

(a) The graph showed the intracellular nPt concentrations in mouse embryonaic fibroblasts (MEF)-wild type cells (white bars), or MEF-Atg5 knockout (K/O) cells (black bars), which were cultured with 50 μg/ml or 100 μg/ml of nPt for 24 h. (b) The graph showed the intracellular nPt concentrations in HchEpC1b-mStrawberry cells (Hch-mSt, left) or HchEpC1b-ATG4B^{C74A} cells (Hch-4B, right) cultured with 25 μg/ml nPt for 24 h in the presence of DMSO (white bars), chlorpromazine (CPZ, 1 μg/ml, light gray bars), methyl-β-cyclodextrin (MβCD, 1mM, dark gray bars), or amiloride (ALR, 100 μM, black bars) . (c, d) The cellular proliferation assay was performed using Hch-mSt cells (c), or Hch-4B (d) in the presence (right) or absence (left) of 100 μg/ml nPt for 24 h. They were treated with DMSO (white bars), CPZ (1 μg/ml, light gray bars), MβCD (1mM, dark gray bars), or ALR (100 μM, black bars). (e) Based on figure 3d, the graph indicated the proportions of nPt concentrations in the HchEpC1b-mStrawberry cells (Hch-mSt, white bars), and HchEpC1b-ATG4B^{C74A} cells (Hch-4B, black bars), which were normalized to those at 0 h as 100%, respectively. *; $p < 0.05$, **; $p < 0.01$, ***; $p < 0.001$.

Supplemental figure 5

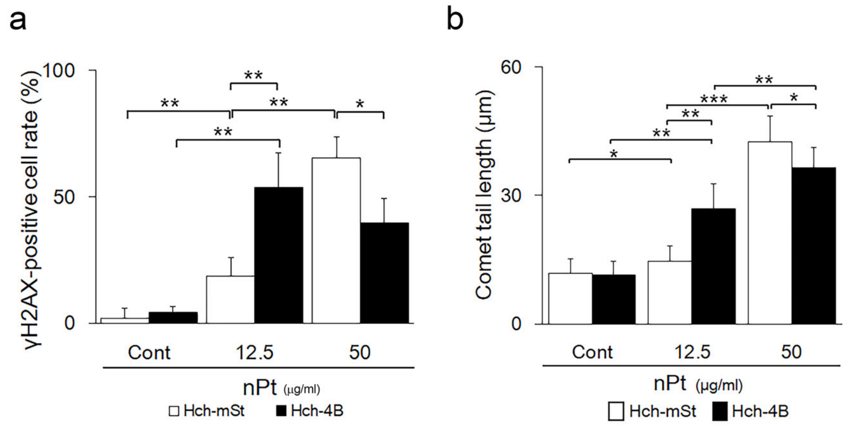

Supplemental Figure 5 Cell death of autophagy-deficient cells by nPt-mediated DNA damage.

(a) The amount of phosphor-γH2AX (p-γH2AX)-positive HchEpC1b-mStrawberry (Hch-mSt, white bars) cells and HchEpC1b-ATG4B^{C74A} (Hch-4B, black bars) cells after culturing with 12.5 μg/ml or 50 μg/ml of nPt for 24 h. (b) The graph showed the comet tail length by comet assay in Hch-mSt or -4B cells with 12.5 μg/ml or 50 μg/ml of nPt for 24 h. Data are expressed as the mean ± S.D..
*; $p < 0.05$, **; $p < 0.01$.

Supplemental figure 6

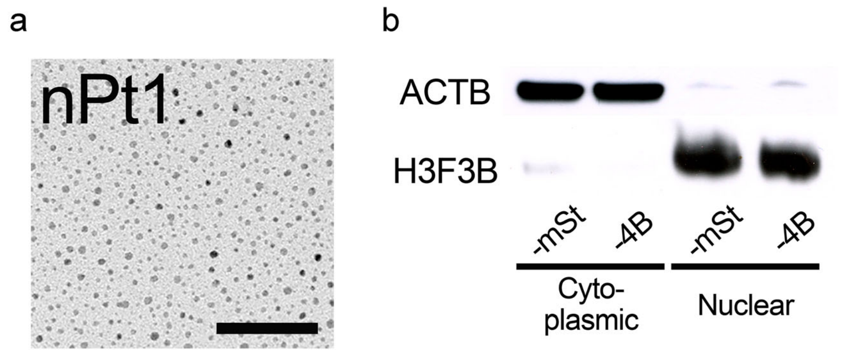

Supplemental Figure 6 Size analysis of nPt1 by TEM and western blots of fractionated lysates

(a) Platinum nanoparticle (nPt1) size was measured by TEM. The average diameter of nPt was 0.63 ± 0.07 nm. (b) Fractionated cytoplasmic and nuclear lysates were obtained from HchEpC1b-mStrawberry cells (-mSt, autophagy-normal) or HchEpC1b-ATG4B^{C74A} cells (-4B, autophagy-deficient), which were cultured with 12.5 μg/ml of nPt for 24 h. Using the lysates, western blots were performed of H3F3B (Histone H3) and ACTB. Scale bar: 50 nm.

Supplemental figure 7 Uncropped gel and blots for figure 1a, 4a, 5d, Supplemental figure 1a, 1b 3b, and 6b.

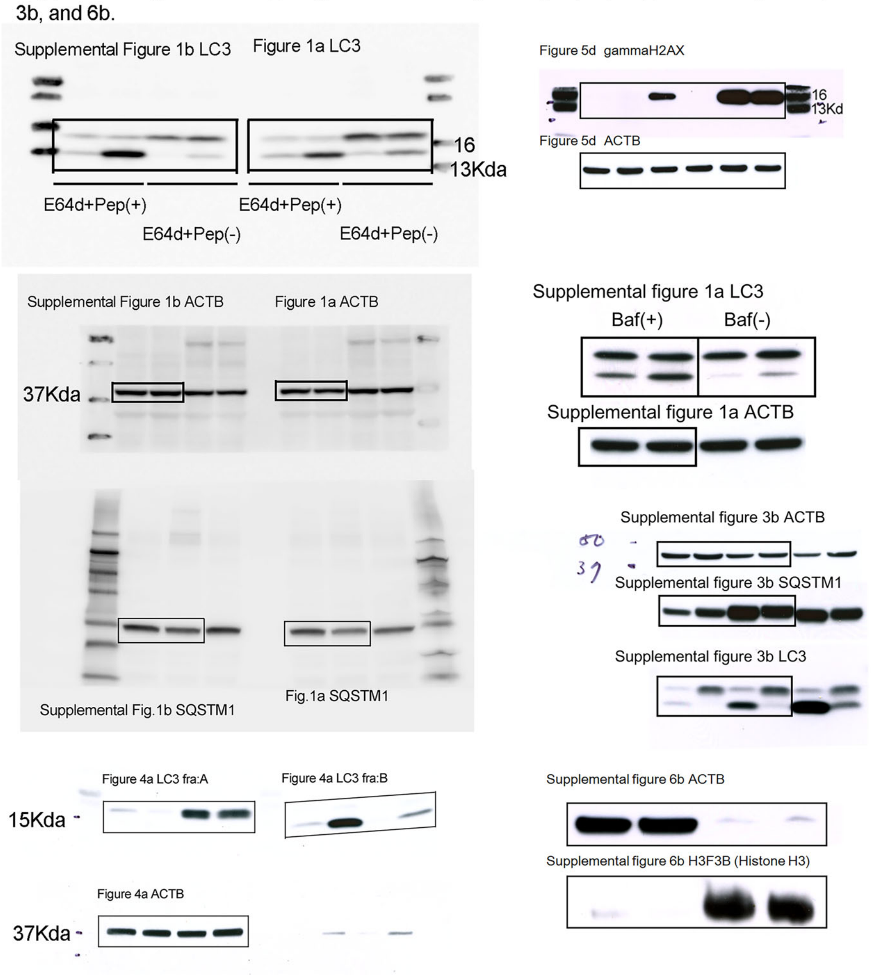

Supplement: Supplementary file 1 — Supplemental figures [file 41598_2019_41927_MOESM1_ESM.pdf]
